# Supplementary material for: Three prime repair exonuclease 1 preferentially degrades the integration-incompetent HIV-1 DNA through favorable kinetics, thermodynamic, structural, and conformational properties
Source: J Biol Chem. 2024 Jun 3;300(7):107438. doi: 10.1016/j.jbc.2024.107438 (PMC11259700; doi:10.1016/j.jbc.2024.107438)
Supplement: Supporting Information [file mmc5.pdf]

## Supplementary Figure 1.
# h-TREX1 activity with U5 unprocessed and processed substrates

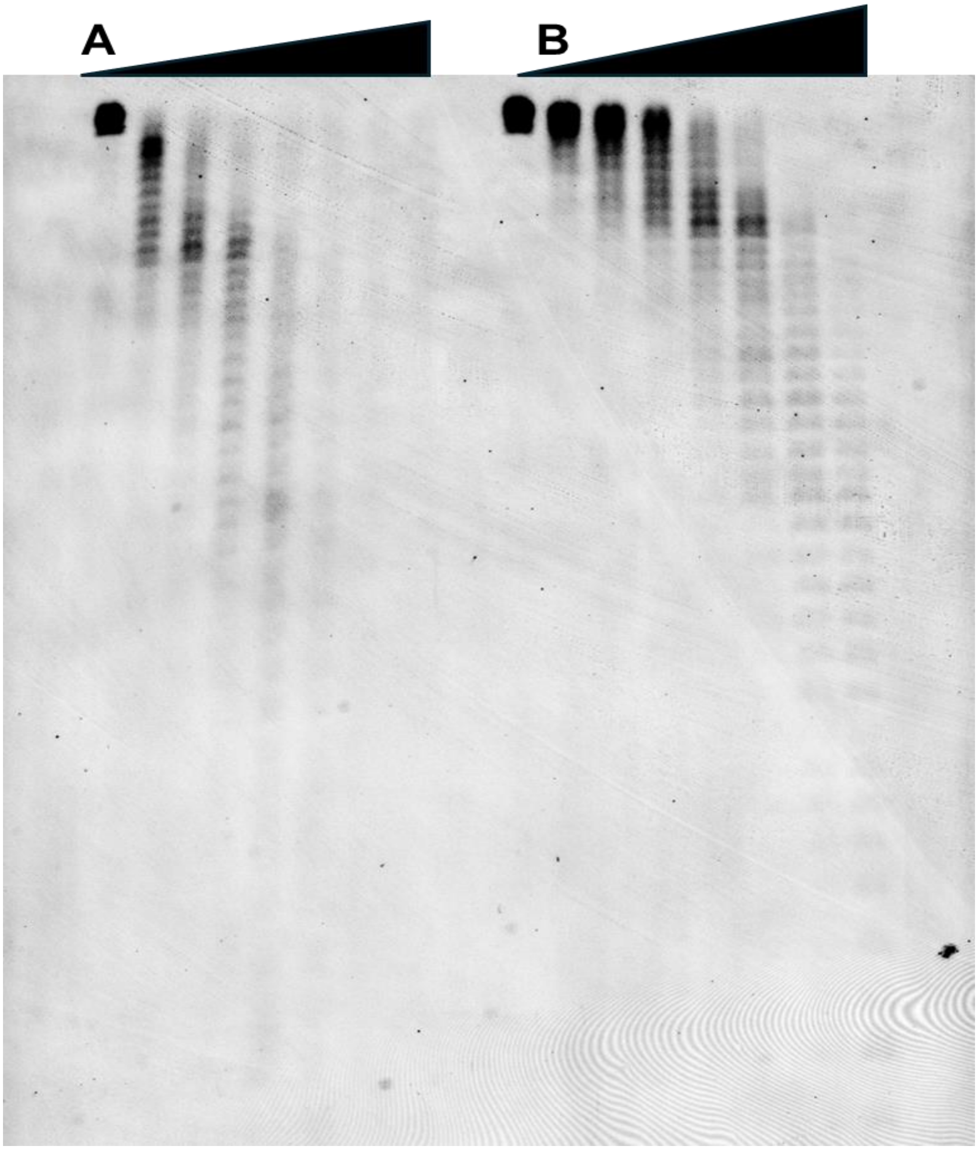


**TREX1 exonuclease assay with longer U5 viral DNA substrate** (A) The exonuclease activity of h-TREX1 was assayed using unprocessed U5 viral DNA (50 base pairs oligonucleotide) at various time points, including 0, 20, 40, 60, 90, 120, 180, and 240 seconds. Time points are indicated by increasing bars positioned above the respective lanes in the figure. (B) The exonuclease activity of h-TREX1 was assayed using processed U5 viral DNA (50 base pairs oligonucleotide) at various time points, including 0, 20, 40, 60, 90, 120, 180, and 240 seconds. Time points are indicated by increasing bars positioned above the respective lanes in the figure.

## Supplementary Figure 2.

## h-TREX1 activity with U3 unprocessed and processed substrates

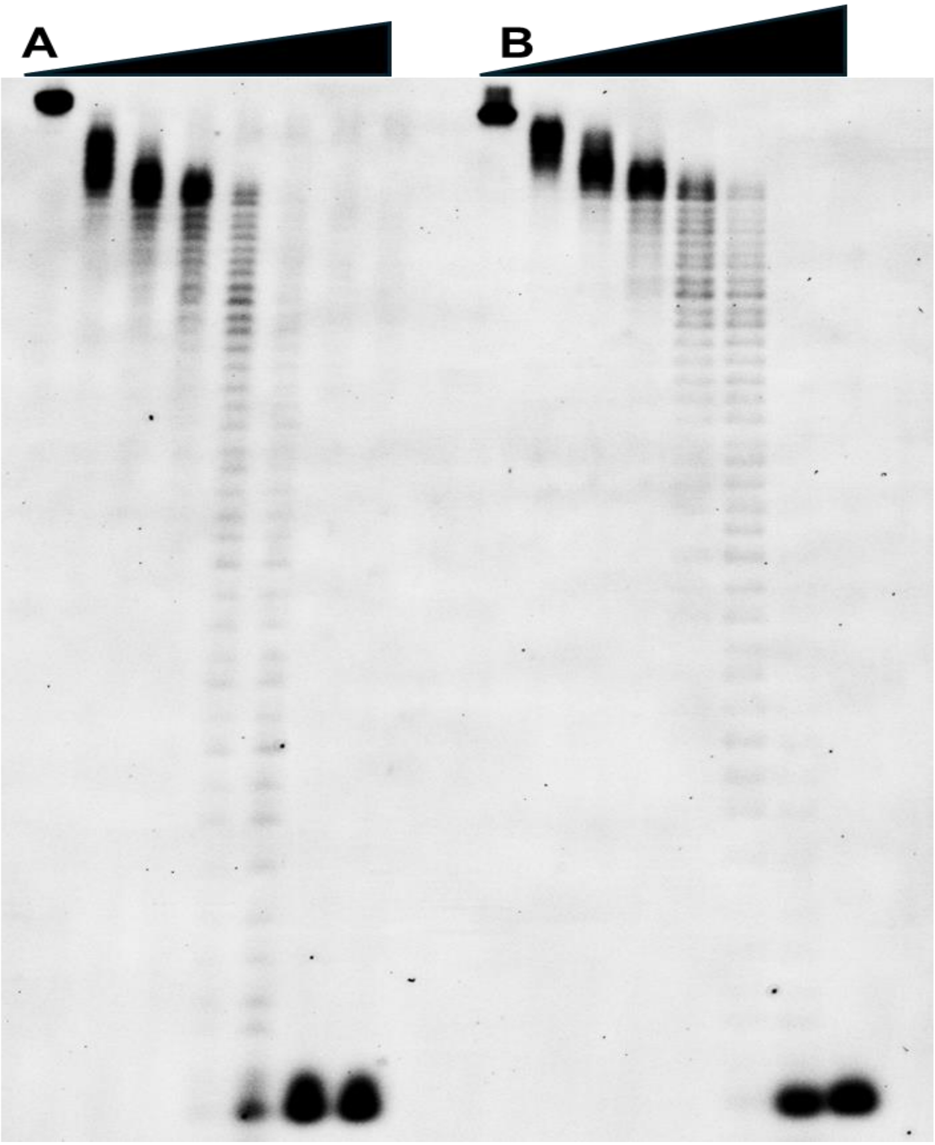


**TREX1 exonuclease assay with longer U3 viral DNA substrate** (A) The exonuclease activity of h-TREX1 was assayed using unprocessed U3 viral DNA (50 base pairs oligonucleotide) at various time points, including 0, 20, 40, 60, 90, 120, 180, and 240 seconds. Time points are indicated by increasing bars positioned above the respective lanes in the figure. (B) The exonuclease activity of h-TREX1 was assayed using processed U3 viral DNA (50 base pairs oligonucleotide) at various time points, including 0, 20, 40, 60, 90, 120, 180, and 240 seconds. Time points are indicated by increasing bars positioned above the respective lanes in the figure.

# Supplementary Figure 3

## Representative Initial velocity curve analysis of h-TREX1 kinetics with U5 unprocessed and processed HIV-1 substrates

**(a)**

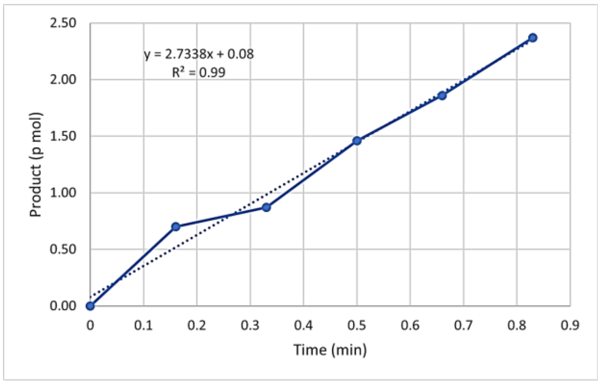


**h-TREX1 with 50 nM U5 unprocessed HIV-1 DNA substrate**

**(d)**

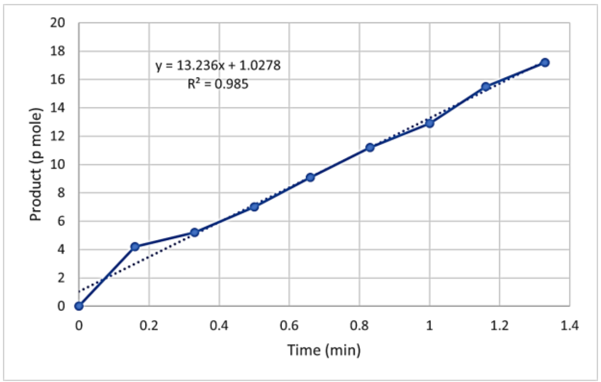


**h-TREX1 with 300 nM U5 unprocessed HIV-1 DNA substrate**

**(b)**

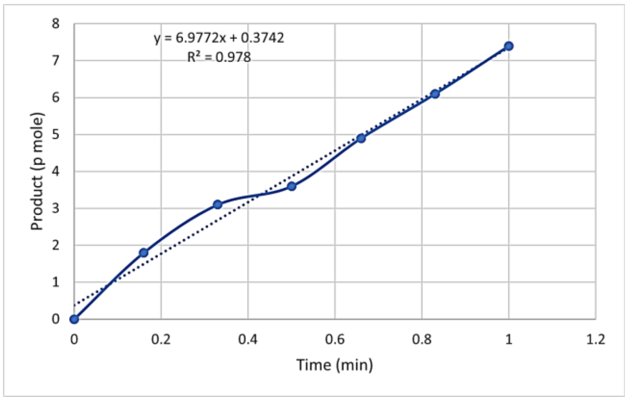


**h-TREX1 with 100 nM U5 unprocessed HIV-1 DNA substrate**

**(e)**

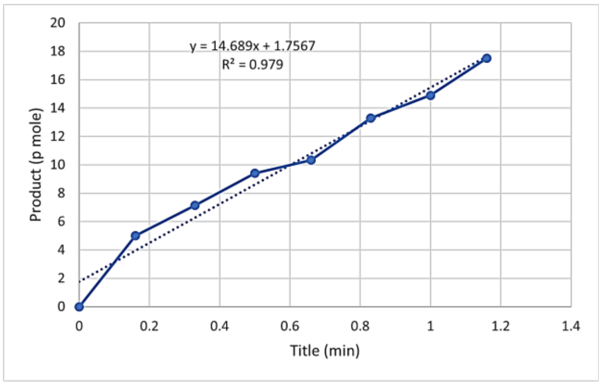


**h-TREX1 with 400 nM U5 unprocessed HIV-1 DNA substrate**

**(c)**

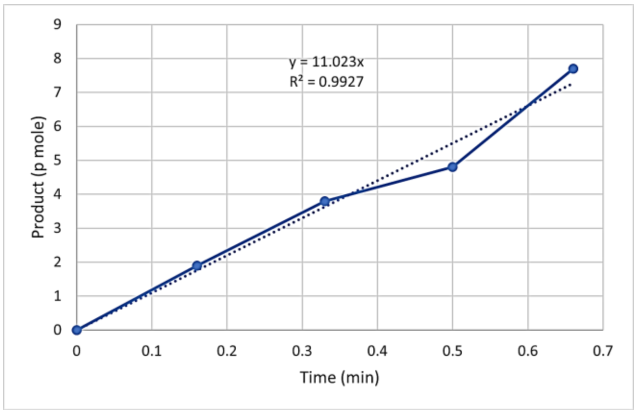


**h-TREX1 with 200 nM U5 unprocessed HIV-1 DNA substrate**

**(f)**

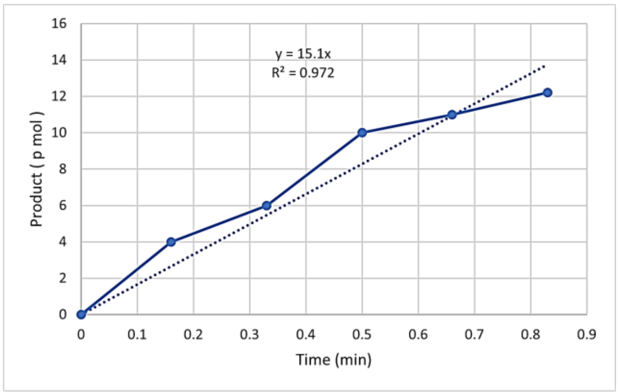


**h-TREX1 with 800 nM U5 unprocessed HIV-1 DNA substrate**

# Supplementary Figure 3

# Representative Initial velocity curve analysis of h-TREX1 kinetics with U5 unprocessed and processed HIV-1 substrates

(g)

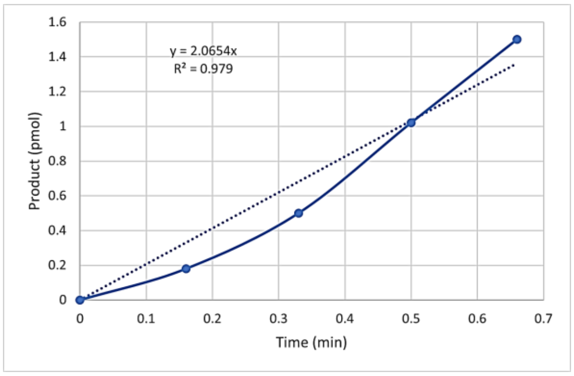


**h-TREX1 with 10 nM U5 Processed HIV-1 DNA substrate**

(i)

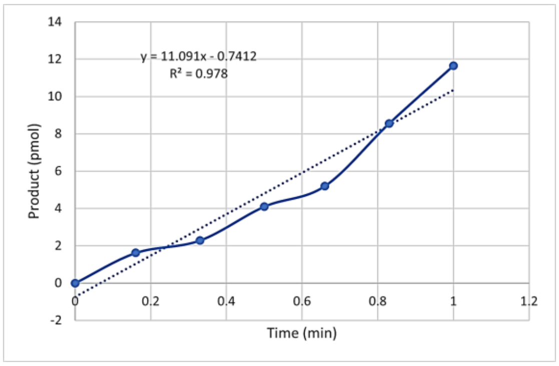


**h-TREX1 with 100 nM U5 Processed HIV-1 DNA substrate**

(h)

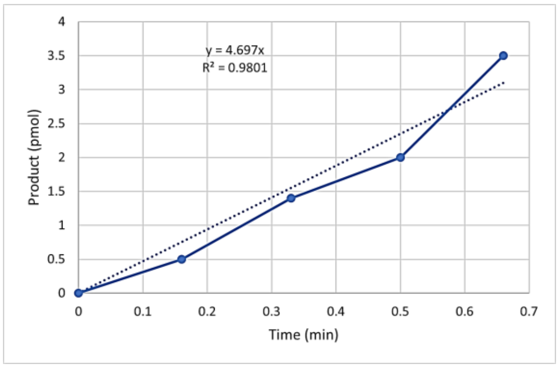


**h-TREX1 with 50 nM U5 Processed HIV-1 DNA substrate**

(j)

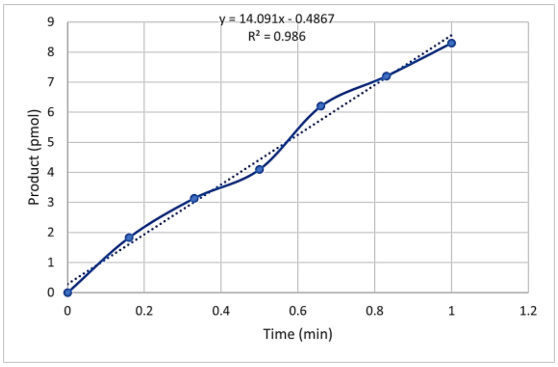


**h-TREX1 with 200 nM U5 Processed HIV-1 DNA substrate**

(k)

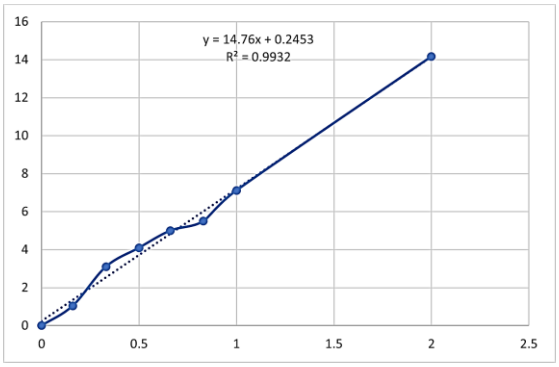


**h-TREX1 with 300 nM U5 Processed HIV-1 DNA substrate**

## Supplementary Figure 4

# Representative Initial velocity curve analysis of m-TREX1 kinetics with U5 unprocessed and processed HIV-1 substrates

**(a)**

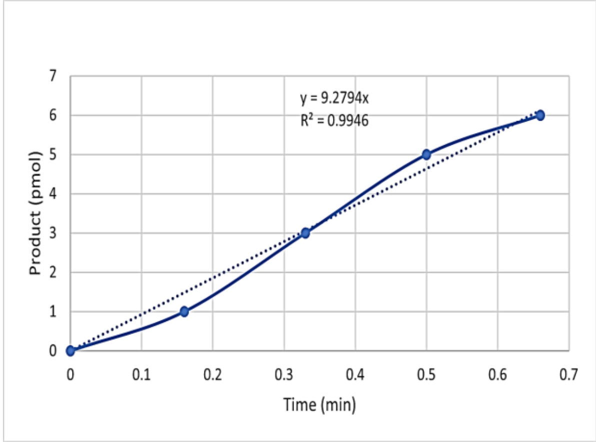


**m-TREX1 with 25 nM U5 Unprocessed HIV-1 DNA substrate**

**(c)**

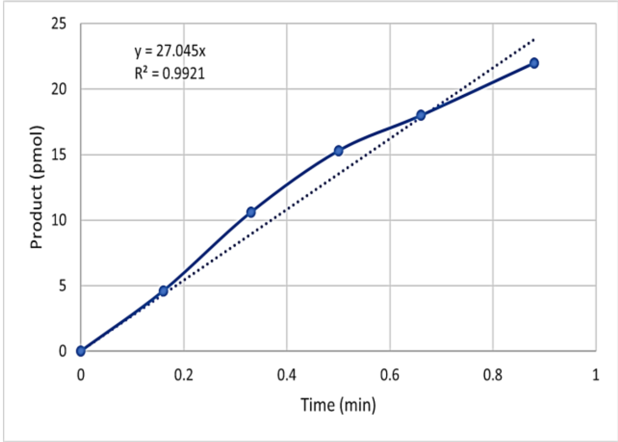


**m-TREX1 with 100 nM U5 Unprocessed HIV-1 DNA substrate**

**(b)**

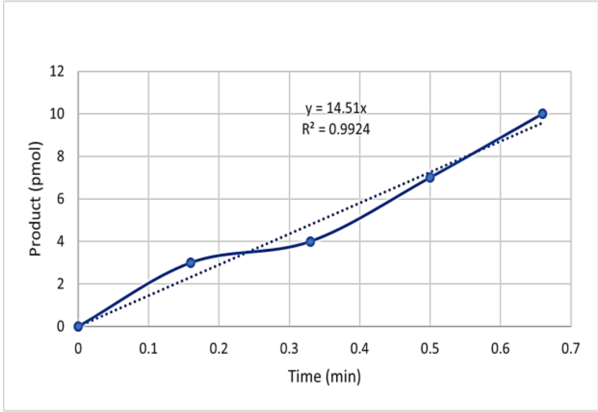


**m-TREX1 with 50 nM U5 Unprocessed HIV-1 DNA substrate**

**(d)**

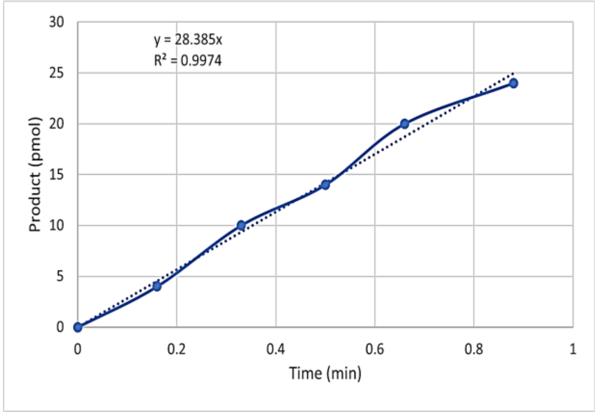


**m-TREX1 with 200 nM U5 Unprocessed HIV-1 DNA substrate**

**(e)**

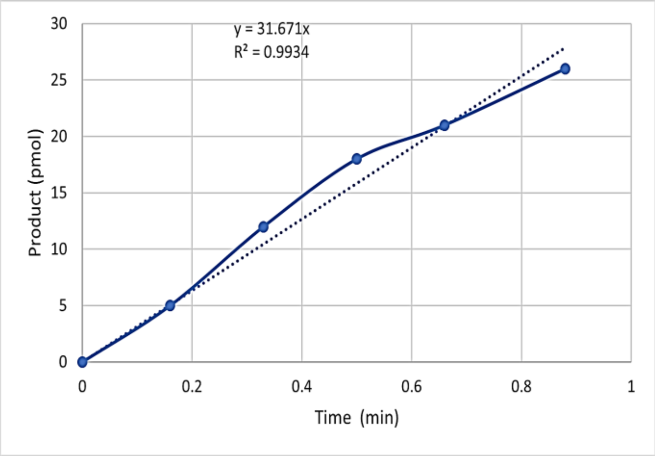


**m-TREX1 with 300 nM U5 Unprocessed HIV-1 DNA substrate**

## Supplementary Figure 4

# Representative Initial velocity curve analysis of m-TREX1 kinetics with U5 unprocessed and processed HIV-1 substrates

**(f)**

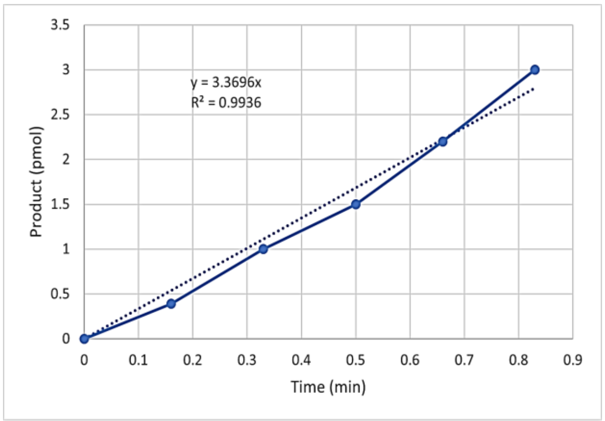


**m-TREX1 with 25 nM U5 Processed HIV-1 DNA substrate**

**(h)**

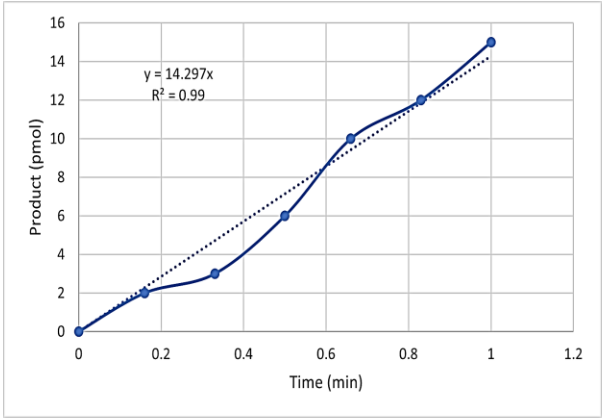


**m-TREX1 with 100 nM U5 Processed HIV-1 DNA substrate**

**(g)**

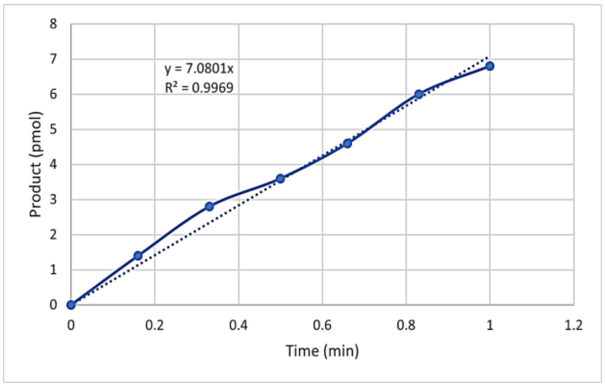


**m-TREX1 with 50 nM U5 Processed HIV-1 DNA substrate**

**(i)**

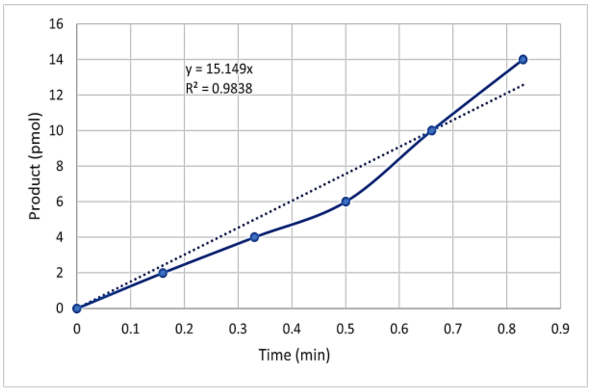


**m-TREX1 with 200 nM U5 Processed HIV-1 DNA substrate**

**(j)**

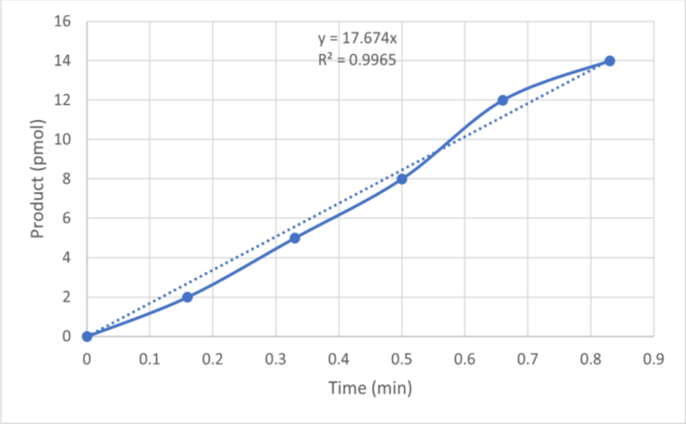


**m-TREX1 with 300 nM U5 Processed HIV-1 DNA substrate**

# Supplementary Figure 5

## Representative Initial velocity curve analysis of h-TREX1 kinetics with U3 unprocessed and processed HIV-1 substrates

**(a)**

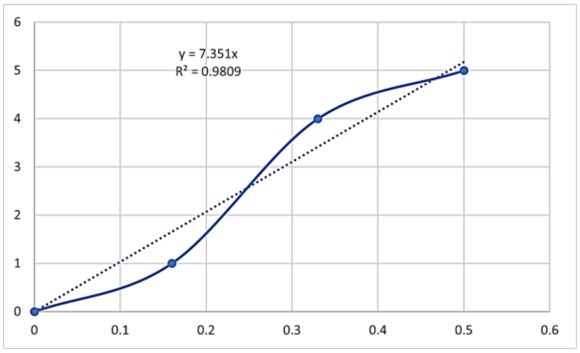


**h-TREX1 with 25 nM U3 Unprocessed HIV-1 DNA substrate**

**(c)**

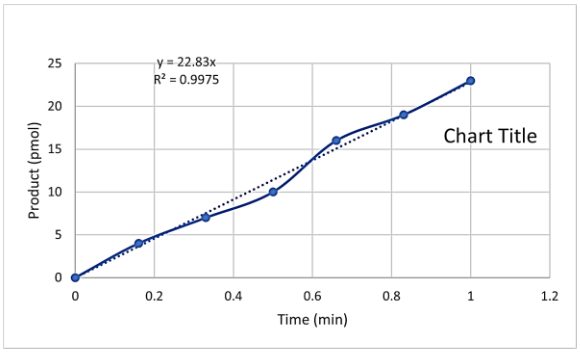


**h-TREX1 with 100 nM U3 Unprocessed HIV-1 DNA substrate**

**(b)**

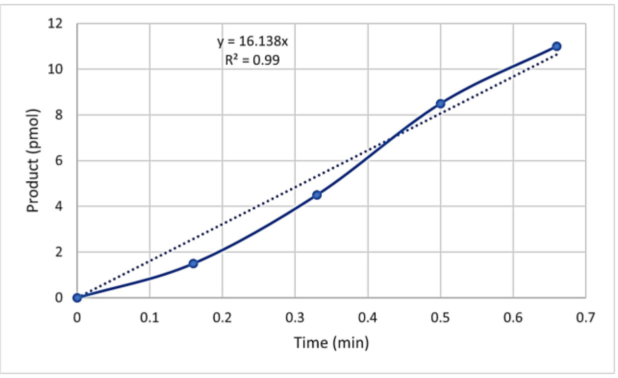


**h-TREX1 with 50 nM U3 Unprocessed HIV-1 DNA substrate**

**(d)**

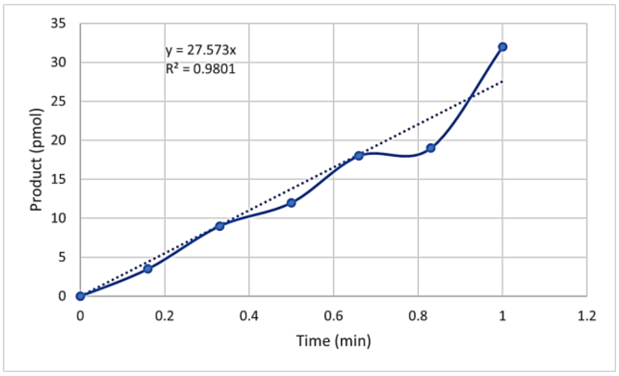


**h-TREX1 with 200 nM U3 Unprocessed HIV-1 DNA substrate**

**(e)**

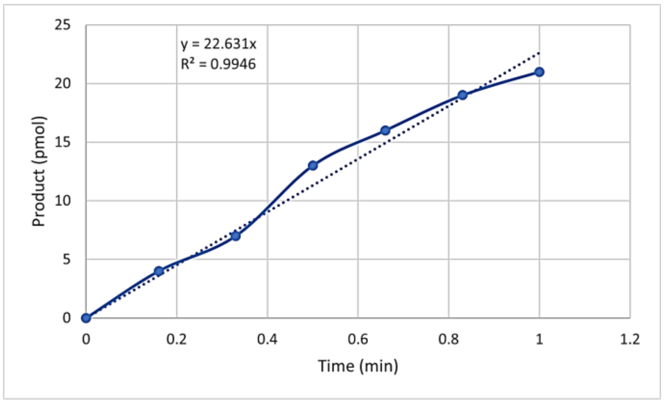


**h-TREX1 with 300 nM U3 Unprocessed HIV-1 DNA substrate**

## Supplementary Figure 5

# Representative Initial velocity curve analysis of h-TREX1 kinetics with U3 unprocessed and processed HIV-1 substrates

**(f)**

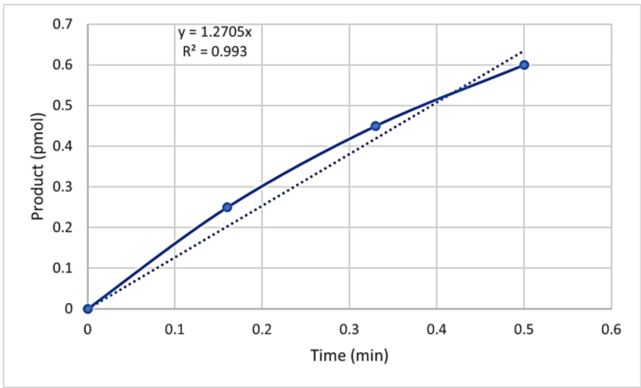


**h-TREX1 with 25 nM U3 Processed HIV-1 DNA substrate**

**(h)**

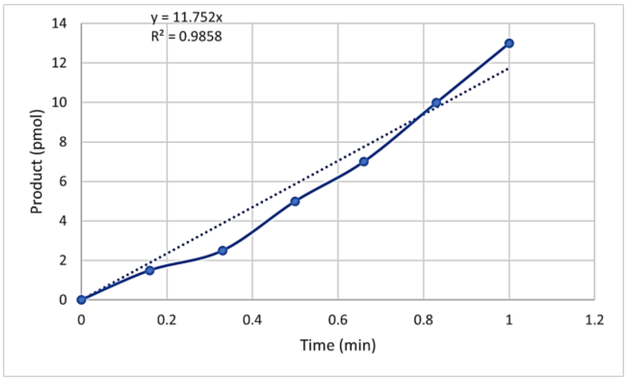


**h-TREX1 with 100 nM U3 Processed HIV-1 DNA substrate**

**(g)**

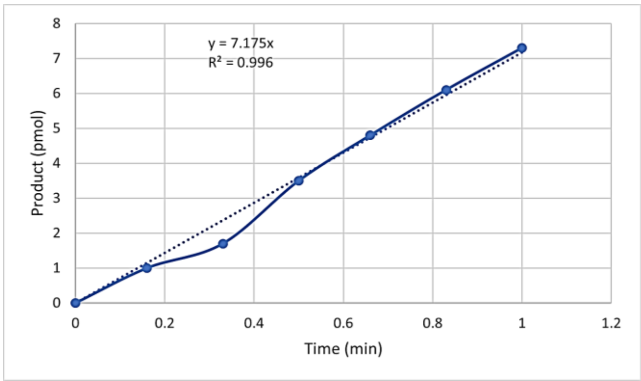


**h-TREX1 with 50 nM U3 Processed HIV-1 DNA substrate**

**(i)**

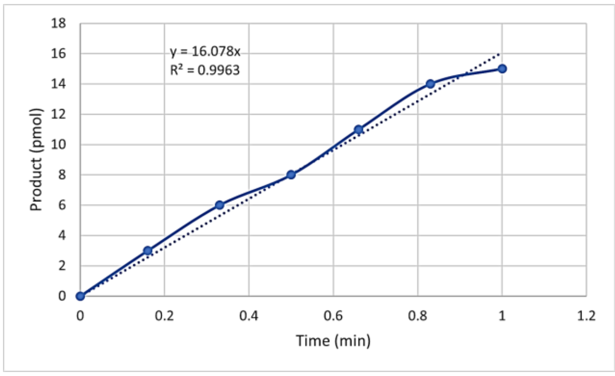


**h-TREX1 with 200 nM U3 Processed HIV-1 DNA substrate**

**(j)**

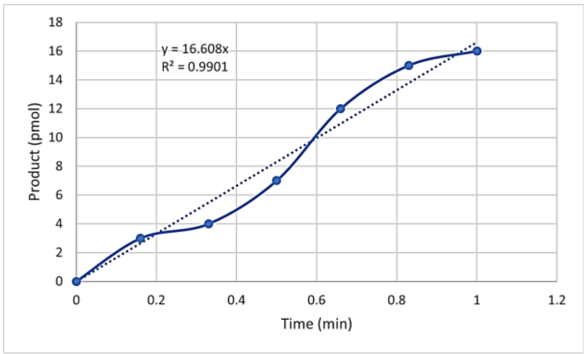


**h-TREX1 with 300 nM U3 Processed HIV-1 DNA substrate**

## Supplementary Figure 6

# Representative Initial velocity curve analysis of m-TREX1 kinetics with U3 unprocessed HIV-1 substrates

(a)

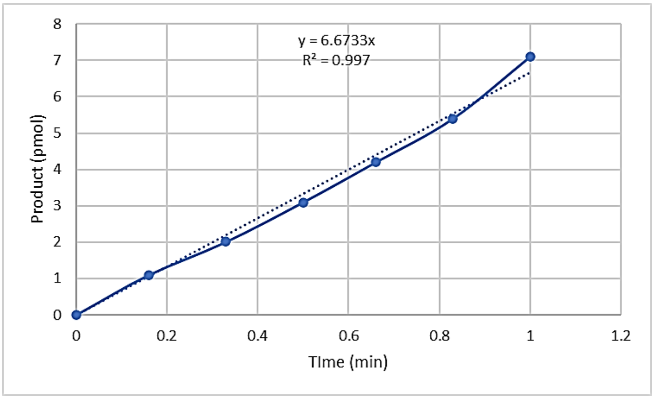


**m-TREX1 with 25 nM U3 Unprocessed HIV-1 DNA substrate**

(b)

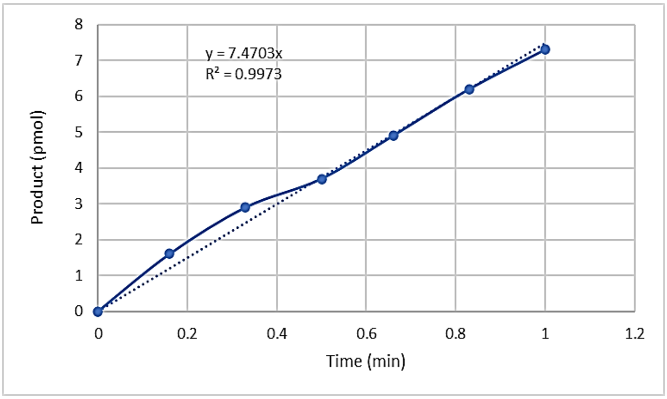


**m-TREX1 with 50 nM U3 Unprocessed HIV-1 DNA substrate**

(c)

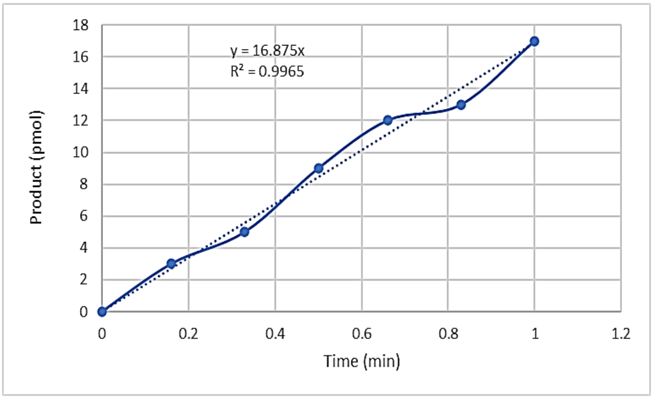


**m-TREX1 with 100 nM U3 Unprocessed HIV-1 DNA substrate**

(d)

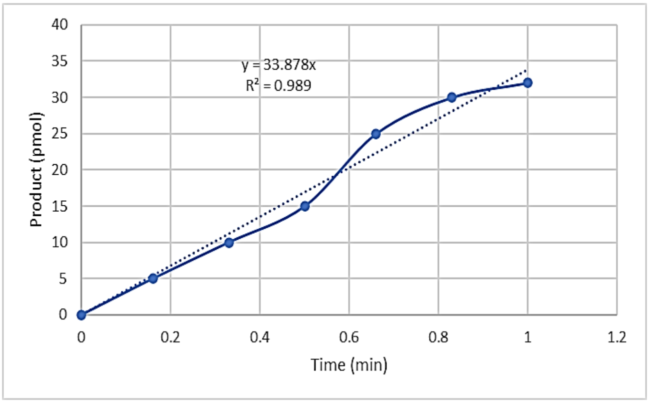


**m-TREX1 with 200 nM U3 Unprocessed HIV-1 DNA substrate**

(e)

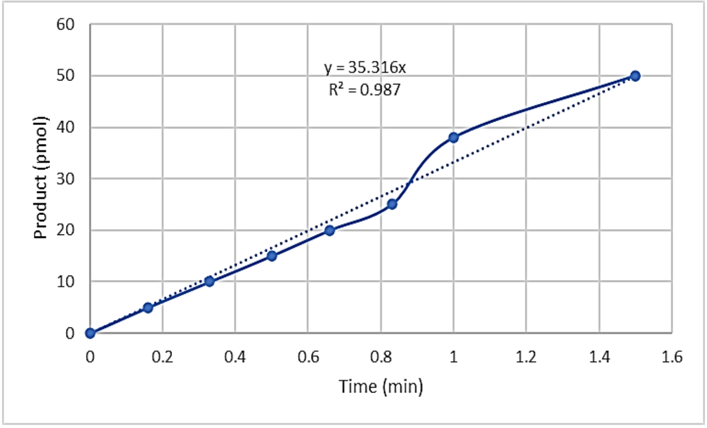


**m-TREX1 with 400 nM U3 Unprocessed HIV-1 DNA substrate**

(f)

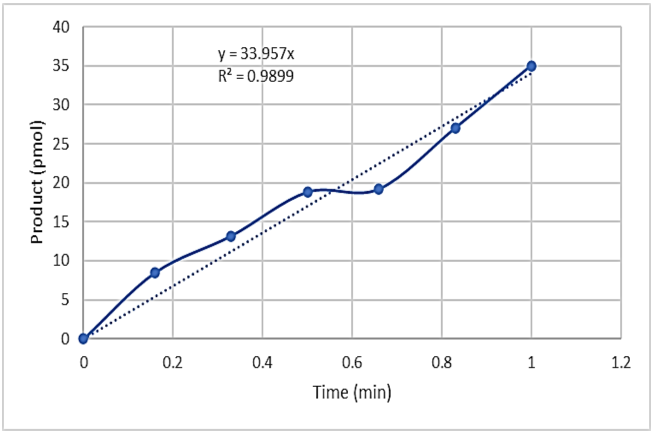


**m-TREX1 with 600 nM U3 Unprocessed HIV-1 DNA substrate**

# Supplementary Figure 6

## Representative Initial velocity curve analysis of m-TREX1 kinetics with U3 processed HIV-1 substrates

(g)

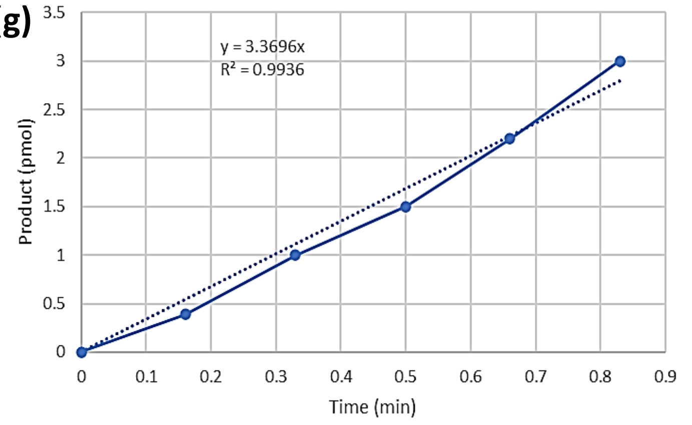


(h)

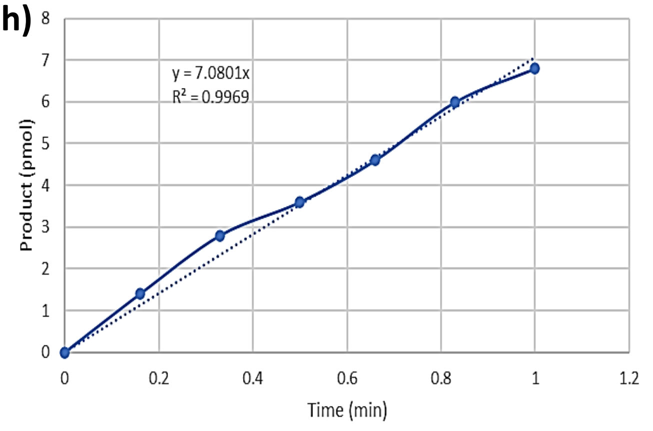


**m-TREX1 with 25 nM U3 Processed HIV-1 DNA substrate**

**m-TREX1 with 50 nM U3 Processed HIV-1 DNA substrate**

(i)

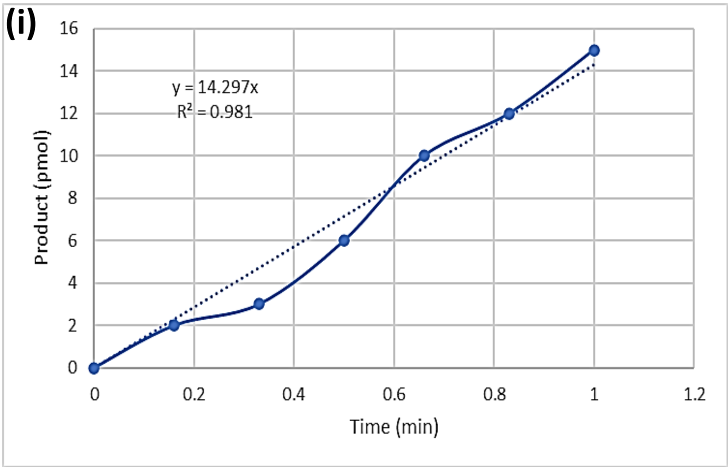


(j)

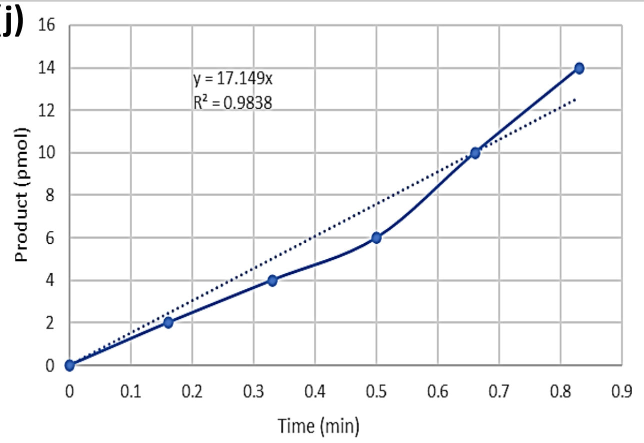


**m-TREX1 with 100 nM U3 Processed HIV-1 DNA substrate**

**m-TREX1 with 200 nM U3 Processed HIV-1 DNA substrate**

(k)

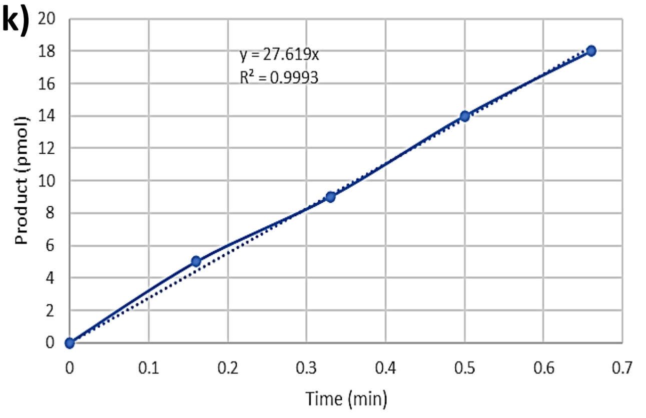


**m-TREX1 with 400 nM U3 Processed HIV-1 DNA substrate**
